# Analysis of separate training and validation radical prostatectomy cohorts identifies 0.25 mm diameter as an optimal definition for “large” cribriform prostatic adenocarcinoma

**DOI:** 10.1038/s41379-022-01009-7

**Published:** 2022-02-10

**Authors:** Emily Chan, Jesse K. McKenney, Sarah Hawley, Dillon Corrigan, Heidi Auman, Lisa F. Newcomb, Hilary D. Boyer, Peter R. Carroll, Matthew R. Cooperberg, Eric Klein, Ladan Fazli, Martin E. Gleave, Antonio Hurtado-Coll, Jeffry P. Simko, Peter S. Nelson, Ian M. Thompson, Maria S. Tretiakova, Dean Troyer, Lawrence D. True, Funda Vakar-Lopez, Daniel W. Lin, James D. Brooks, Ziding Feng, Jane K. Nguyen

**Affiliations:** 1 Department of Pathology, University of California San Francisco (UCSF), San Francisco, CA USA; 2 Robert J. Tomsich Institute of Pathology and Laboratory Medicine, Cleveland Clinic, Cleveland, OH USA; 3 Canary Foundation, Palo Alto, CA USA; 4 Department of Quantitative Health Sciences, Lerner Research Institute, Cleveland Clinic, Cleveland, OH USA; 5 Fred Hutchinson Cancer Research Center, Seattle, WA USA; 6 University of Washington Medical Center, Seattle, WA USA; 7 Department of Urology, University of California San Francisco (UCSF), San Francisco, CA USA; 8 Glickman Urological and Kidney Institute, Cleveland Clinic, Cleveland, OH USA; 9 University of British Columbia, Vancouver, BC Canada; 10 CHRISTUS Medical Center Hospital, San Antonio, TX USA; 11 Eastern Virginia Medical School, Norfolk, VA USA; 12 Department of Pathology, UT Health, San Antonio, TX USA; 13 Stanford University Medical Center, Stanford, CA USA

**Keywords:** Prostate cancer, Tumour biomarkers

## Abstract

Cribriform growth pattern is well-established as an adverse pathologic feature in prostate cancer. The literature suggests “large” cribriform glands associate with aggressive behavior; however, published studies use varying definitions for “large”. We aimed to identify an outcome-based quantitative cut-off for “large” vs “small” cribriform glands. We conducted an initial training phase using the tissue microarray based Canary retrospective radical prostatectomy cohort. Of 1287 patients analyzed, cribriform growth was observed in 307 (24%). Using Kaplan–Meier estimates of recurrence-free survival curves (RFS) that were stratified by cribriform gland size, we identified 0.25 mm as the optimal cutoff to identify more aggressive disease. In univariable and multivariable Cox proportional hazard analyses, size >0.25 mm was a significant predictor of worse RFS compared to patients with cribriform glands ≤0.25 mm, independent of pre-operative PSA, grade, stage and margin status (*p* < 0.001). In addition, two different subset analyses of low-intermediate risk cases (cases with Gleason score ≤ 3 + 4 = 7; and cases with Gleason score = 3 + 4 = 7/4 + 3 = 7) likewise demonstrated patients with largest cribriform diameter >0.25 mm had a significantly lower RFS relative to patients with cribriform glands ≤0.25 mm (each subset *p* = 0.004). Furthermore, there was no significant difference in outcomes between patients with cribriform glands ≤ 0.25 mm and patients without cribriform glands. The >0.25 mm cut-off was validated as statistically significant in a separate 419 patient, completely embedded whole-section radical prostatectomy cohort by biochemical recurrence, metastasis-free survival, and disease specific death, even when cases with admixed Gleason pattern 5 carcinoma were excluded. In summary, our findings support reporting cribriform gland size and identify 0.25 mm as an optimal outcome-based quantitative measure for defining “large” cribriform glands. Moreover, cribriform glands >0.25 mm are associated with potential for metastatic disease independent of Gleason pattern 5 adenocarcinoma.

## Introduction

Recent consensus statements by the two major urologic pathology societies (International Society of Urologic Pathology, ISUP; and Genitourinary Pathology Society, GUPS) both recommend including the presence or absence of cribriform glands in the pathology report based on strong evidence that cribriform architecture is associated with adverse clinical outcomes^1,2^. However, a number of additional size descriptors including “small” or “simple,” and “large” or “expansile,” have also been used in the literature to subclassify cribriform glands. Although early studies failed to find any correlation between cribriform gland size with other adverse prognostic features or outcomes^3,4^, recent studies show that “large” cribriform glands are a significant adverse prognostic factor^5–8^. These differences are likely due, at least in part, to the use of different qualitative definitions. Definitions for “large” cribriform glands have included size relative to adjacent preexistent normal glands and more than 12 luminal spaces, leading to the lack of a published consensus definition. Moreover, in our experience these criteria can be difficult to apply in some cases and may suffer from interobserver variability, particularly in needle core biopsies. In this study, we sought to identify a clinical outcome-based quantitative definition for “large” cribriform glands using an objective, easily recordable measure for size: diameter of largest cribriform gland.

## Methods

### Training cohort

We used a multi-institutional prostate cancer tissue microarray, the Canary Prostate Cancer Tissue Microarray (CPCTM), which was constructed from radical prostatectomy (RP) samples from 1995 to 2004 to evaluate the prognostic value of tissue biomarkers in men diagnosed with prostate cancer^9^. The CPCTM included three 1 mm cores of cancer tissue obtained from the highest grade cancer pattern in each patient’s prostatectomy. Centralized grading of the TMA cores was previously performed (by JKM);^6^ each patient’s highest-grade core was the grade used in the analyses of this study. Patients were included in the current study if at least one core on histologic review included cancer tissue and follow-up data was completed. The following subsets were also analyzed to evaluate the significance of cribriform morphology and cribriform size in a “low-intermediate” risk population: patients who had up to GS 3 + 4 = 7 tumor in their highest grade core (Subset: Gleason score (GS) ≤ 3 + 4 = 7; combined Grade Groups 1 and 2), patients who had GS 3 + 4 = 7 tumor in their highest grade core (Subset: GS = 3 + 4 = 7; Grade Group 2 only); and patients who had either GS 3 + 4 = 7 or 4 + 3 = 7 tumor in their highest grade core (Subset: GS = 3 + 4 = 7/4 + 3 = 7; combined Grade Groups 2 and 3).

**Fig. 1:**
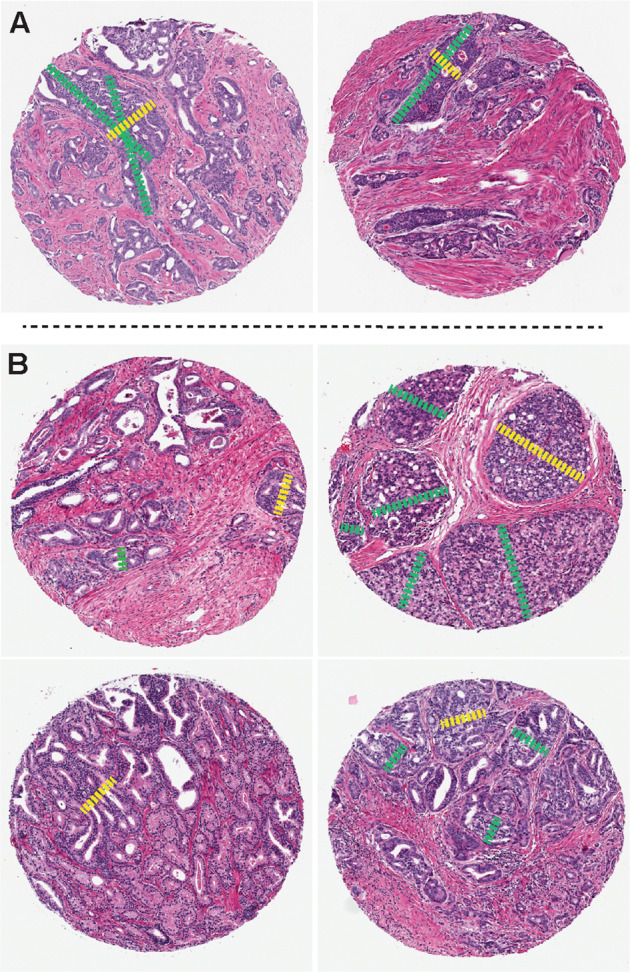
Sample histologic images of training cohort TMA spots containing cribriform glands and how cribriform size is measured. **A** Yellow dashed lines indicate how cribriform gland size is measured using longest cross-sectional distance rather than longitudinal measurement (green dashed line), which can overestimate tangentially sectioned and branching glands. **B** Yellow dashed lines indicate diameter of the largest cribriform gland in each spot (with smaller cribriform gland diameters indicated in green dashed line).

One genitourinary pathologist (EC) evaluated the 5-micron TMA sections stained with hematoxylin and eosin for the presence/absence of cribriform glands. Cribriform glands were defined as any solid nest of tumor cells containing at least 3 “punched out” luminal spaces. The range of cribriform glands seen and how size was measured is illustrated in Fig. 1. The diameter of the largest cribriform gland was obtained by measuring the longest cross-sectional distance of the cribriform glands identified in each core. It is important to note that size was obtained using cross-sectional distance, rather than longitudinal distance (indicated by yellow dashed lines as opposed to green dashed lines in Fig. 1A), to avoid measuring a tangentially sectioned or branching cribriform gland. If multiple cribriform glands were present, the diameter of the largest cribriform gland in each core was measured (yellow dashed lines in Fig. 1B). Cribriform glands with a solid component were measured across their entire diameter. Simple glomerulation glands (tumor glands with intraluminal cell clusters with only one connection to the gland wall and occupying less than half of the inner gland surface) were not considered cribriform^10^. The largest diameter recorded amongst the three tumor cores analyzed for each patient was used as the “cribriform size” for further statistical analysis. As per the original Canary study and other published series^2,6,11–13^, intraductal carcinoma of the prostate and invasive cribriform glands were combined for analysis due to inherent problems in their distinction and their similar prognostic significance.

The primary endpoint was defined as post-surgery recurrence-free survival (RFS) from date of RP. RFS was defined as absence of PSA (biochemical) recurrence, local recurrence, initiation of salvage therapy, prostate cancer metastases, or death from prostate cancer. Events were scored at the earliest date an event occurred after surgery. Biochemical recurrence was defined as a single PSA measurement of 0.2 ng/mL or greater, more than 8 weeks after RP.

Fisher’s Exact Test, Pearson Chi-Squared Test, Wilcoxon Test or Kruskal–Wallis Test were used to assess the association between presence of cribriform glands and other clinical-pathologic variables including age at RP, pre-operative PSA (median log transformed), grade, extra-capsular extension, seminal vesicle invasion and positive surgical margins. Kaplan–Meier (KM) method was used to estimate survival when considering various cutoffs (median, quartile, and semi-interquartile) of the size of the largest cribriform gland to predict RFS. Patient characteristics, presence of a cribriform gland and the size of the largest cribriform gland were evaluated as predictors of RFS in both univariable and multivariable Cox proportional hazards models. All tests were two-sided and *p* values of 0.05 or less were considered statistically significant. Statistical analysis was carried out using SAS version 9 (SAS Institute, Cary, NC). Kaplan–Meier plots were generated using R [R Core Team (2019). R Foundation for Statistical Computing, Vienna, Austria. URL https://www.R-project.org/].

### Validation cohort

This separate RP cohort from the Cleveland Clinic has been reported previously and has no patient overlap with the training cohort^14,15^. In brief, the cohort sampled from a larger set of 2641 clinical T1/T2 patients with PCa treated by RP at the Cleveland Clinic from 1987 to 2004. All patients with clinical recurrence were selected (local recurrence or distant metastasis, *n* = 127), together with a sampling of nonrecurrent patients (*n* = 374), using an established stratified cohort sampling method with a 1:3 ratio of recurrent to nonrecurrent patients. Biochemical recurrence was defined as a rising PSA measurement reaching 0.2 ng/mL or greater, more than 8 weeks after RP. Follow-up data was continuously updated from the time of the original publication through subsequent clinic visits, telephone calls, and semi-annual follow-up letters obtained through August 1, 2019 (~10 years after the previously reported cut-off)^14^. Multiple data reviews and quality checks were performed to ensure fidelity of the dataset. All RPs were submitted entirely for histologic review and were re-evaluated by a genitourinary pathologist (JKN) for conventional GS/Grade Group, the presence of cribriform glands, the size of cribriform glands >0.25 mm as defined in test cohort, the presence of conventional Gleason pattern 5 (using latest grading criteria from the 2014 ISUP Consensus Conference on Gleason Grading Patterns published in 2016)^16^, and pathologic stage. Three categories were evaluated: Group A: carcinomas with no cribriform glands or cribriform ≤0.25 mm; Group B: cribriform >0.25 mm without Gleason pattern 5; and Group C: any Gleason pattern 5 present. Tumors with any Gleason pattern 5 (Group C) were excluded from the >0.25 mm cribriform group (Group B) and evaluated separately to control for conventional non-cribriform high-risk patterns driving clinical significance.

Survival functions for each Group A–C were estimated via Kaplan–Meier analysis for each outcome of interest. Survival distributions of the cribriform groups were compared in a pairwise manner with log-rank tests. Pairwise log-rank test *p* values were adjusted for multiple comparisons using the Benjamini–Hochberg procedure. Where applicable, median survival times and their confidence intervals (CIs) were also reported.

## Results

### Training cohort

**Fig. 2:**
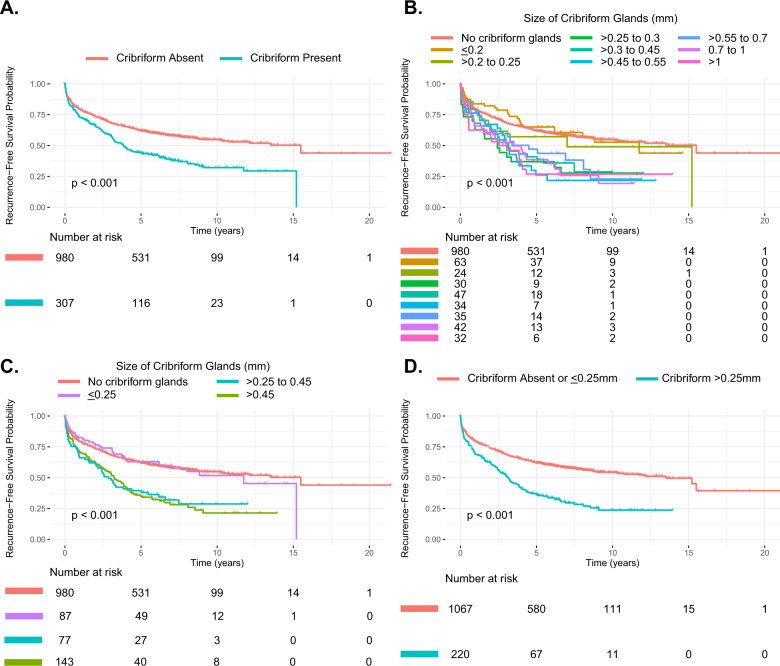
Kaplan–Meier curves for recurrence-free survival in full training cohort. **A** Cribriform present vs absent. **B**–**C** Using various incremental cut-offs for cribriform size. **D** Cribriform size ≤0.25 mm vs >0.25 mm.

In total, 1287 patients of the original 1328 (97%) cohort met inclusion criteria. Of these, cribriform pattern was scored as “present” in 307 (24%) patients and “absent” in 980 (76%) (Table 1). In univariable Cox proportional hazards analysis, patients with any cribriform gland present had lower RFS compared to those without any cribriform glands (Hazard Ratio (HR) 1.66; 95% CI 1.39; 1.98, *p* < 0.001) (Table 2, Fig. 2A). When tested for association with other clinical and pathological features at RP (Table 1), we found that presence of cribriform glands was significantly associated with higher median age at surgery (63.5 v. 62.0 years; *p* = 0.015, Wilcoxon Test) and median log pre-operative PSA (2.0 v. 1.8; *p* < 0.001, Wilcoxon Test). Cribriform glands were also associated with higher Gleason grade (*p* < 0.001, Pearson Chi-Squared Test), extra-capsular extension (40% vs 27%; *p* < 0.001, Fisher’s Exact Test) and seminal vesicle invasion (10% v. 6%; *p* = 0.008, Fisher’s Exact Test). There was no correlation between cribriform glands and positive surgical margins.

**Table 1:** Summary of association between cribriform pattern and clinical-pathologic variables at radical prostatectomy for training cohort.

	Cribriform pattern				*P* value^a^
	*Absent*	* Present*			
Variable		*Total*	*Small (*≤*0.25* *mm)*	* Large (>0.25* *mm)*	
Entire cohort (*n* = 1287)	*980 (76%)*	*307 (24%)*	*87 (7%)*	*220 (17%)*	
Median age	62.5	63.5	64	63	0.015^b^/0.051^c^
Pre-operative PSA	1.8	2	1.8	2.1	<0.001^b^/<0.001^c^
Gleason grade					<0.001^d^/<0.001^d^
3 + 3	496/526 (94%)	30/526 (6%)	13/526 (2%)	17/526 (3%)	
3 + 4	268/341 (79%)	73/341 (21%)	36/341 (11%)	37/341 (11%)	
4 + 3	68/155 (44%)	87/155 (56%)	21/155 (14%)	66/155 (43%)	
4 + 4 and higher	60/172 (35%)	112/172 (65%)	14/172 (8%)	98/172 (57%)	
Extra-capsular extension	261/964 (27%)	121/304 (40%)	26/86 (30%)	95/218 (43%)	<0.001^e^/<0.001^d^
Positive surgical margins	300/876 (34%)	91/275 (33%)	21/75 (28%)	70/200 (35%)	0.771^e^/0.518^d^
Seminal vesicle invasion	55/969 (6%)	31/301 (10%)	5/86 (6%)	26/215 (12%)	0.008^e^/0.003^d^

^a^*p* value is provided for “cribriform absent vs present (total)”/“cribriform absent vs present small vs present large”.

^b^Wilcoxon Test.

^c^Kruskal–Wallis Test.

^d^Pearson Chi-Squared Test.

^e^Fisher’s Exact Test.

**Table 2:** Univariable Cox proportional hazards models for training cohort.

Population	Variable	Comparison	Hazard ratio (95% CI)	*P* value	No. Event/Censored/Total Patients
Study population	Cribriform gland	*Present v. Absent*	1.66 (1.39, 1.98)	<0.001^a^	588/699/1287
	Size of largest cribriform gland	*≤0.25* *mm* *v. >0.25* *mm*	1.99 (1.65, 2.40)	<0.001^a^	588/699/1287
Subset: GS ≤ 3 + 4 = 7	Cribriform gland	*Present v. Absent*	1.27 (0.94, 1.71)	0.126	343/524/867
	Size of largest cribriform gland	*≤0.25* *mm* *v. >0.25* *mm*	1.71 (1.18, 2.48)	0.004^a^	343/524/867
Subset: GS = 3 + 4 = 7/4 + 3 = 7	Cribriform gland	*Present v. Absent*	1.17 (0.90, 1.51)	0.247	253/243/496
	Size of largest cribriform gland	*≤0.25* *mm* *v. >0.25* *mm*	1.52 (1.14, 2.02)	0.004^a^	253/243/496

^a^Statistically significant; *p* value < 0.05.

Amongst the patients with cribriform glands, the size of the largest cribriform gland in each patient ranged from 0.05 mm to at least 1 mm (median size: 0.45 mm, interquartile range: 0.45 mm; 1 mm core size maximum dimension). In a univariable proportional-hazards model to describe the effect of cribriform gland size on outcomes, we found that larger cribriform glands were associated with lower RFS (risk ratio 1.49 for every 0.1 mm increase in size of the largest cribriform gland). Therefore, to determine an outcome-based cutoff of what should be considered a “large” cribriform gland, we used the Kaplan–Meier method to estimate survival when considering incremental cutoffs for cribriform gland size. We found that a 0.25 mm cut-off separated patients into two distinct risk groups: patients with cribriform glands ≤0.25 mm had a risk profile similar to that of patients with no cribriform glands, whereas patients with cribriform glands >0.25 mm all demonstrated a similarly lower RFS (Fig. 2B–C).

We then performed additional analysis to evaluate >0.25 mm as a cut-off for “large” cribriform glands. Cribriform glands >0.25 mm in greatest diameter were seen in 72% (220/307) of patients with cribriform glands present (17% of total cohort, 220/1287, Table 1). On univariable Cox proportional hazards analysis, presence of cribriform glands >0.25 mm was significantly associated with lower RFS (HR 1.99, 95% CI 1.65; 2.40, *p* < 0.001, Table 2, Fig. 2D); and on multivariable Cox analysis, presence of cribriform glands with largest diameter >0.25 mm was also associated with lower RFS, independent of pre-operative PSA, GS, extra-capsular extension, positive surgical margins, and seminal vesicle invasion at RP (Table 3).

**Table 3:** Multivariable Cox proportional hazards models for training cohort.

Population	Variable	Comparison	Hazard ratio (95% CI)	*P* value
Study population (*N* = 989; 468 events, 521 censored)	Size of largest cribriform gland	*No glands or glands* ≤*0.25* *mm* *v. >0.25* *mm*	1.63 (1.31, 2.02)	<0.001^a^
	Gleason Score 7 (Grade Group 2–3)	*Gleason Score 7* *v. Gleason Score 6*	1.32 (1.07, 1.62)	0.008^a^
	Gleason Score ≥ 8 (Grade Group 4–5)	*Gleason Score* ≥ *8* *v. Gleason Score* ≤ *6*	1.33 (0.98, 1.81)	0.066
	Pre-operative PSA	*1 unit change*	1.49 (1.29, 1.73)	<0.001^a^
	Extra-prostatic extension	*Positive v. Negative*	1.30 (1.06, 1.59)	0.012^a^
	Seminal vesicle invasion	*Positive v. Negative*	2.12 (1.58, 2.83)	<0.001^a^
	Surgical margins	*Positive v. Negative*	1.50 (1.23, 1.83)	<0.001^a^
Subset: GS ≤ 3 + 4 = 7 (*N* = 661; 272 events, 389 censored)	Size of largest cribriform gland	*No glands or glands* ≤*0.25* *mm* *v. >0.25* *mm*	1.61 (1.08, 2.39)	0.018^a^
	Pre-operative PSA	*1 unit change*	1.41 (1.17, 1.71)	<0.001^a^
	Extra-prostatic extension	*Positive v. Negative*	1.28 (0.98, 1.68)	0.067
	Seminal vesicle invasion	*Positive v. Negative*	2.44 (1.59, 3.74)	<0.001^a^
	Surgical margins	*Positive v. Negative*	1.76 (1.37, 2.27)	<0.001^a^
Subset: GS = 3 + 4 = 7/4 + 3 = 7 (*N* = 398; 203 events, 195 censored)	Size of largest cribriform gland	*No glands or glands* ≤*0.25* *mm* *v. >0.25* *mm*	1.48 (1.07, 2.04)	0.017^a^
	Pre-operative PSA	*1 unit change*	1.54 (1.24, 1.92)	<0.001^a^
	Extra-prostatic extension	*Positive v. Negative*	1.28 (0.98, 1.68)	0.067
	Seminal vesicle invasion	*Positive v. Negative*	2.37 (1.56, 3.61)	<0.001^a^
	Surgical margins	*Positive v. Negative*	1.47 (1.08, 2.00)	0.013^a^

^a^Statistically significant; *p* value < 0.05.

**Fig. 3:**
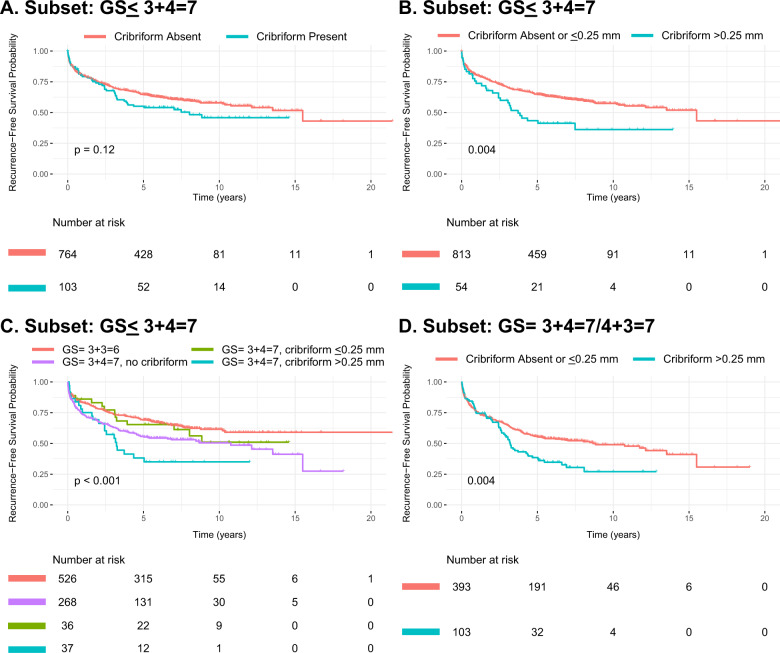
Kaplan–Meier curves for recurrence-free survival in training cohort: subset analysis. **A**–**C** GS ≤ 3 + 4 = 7 subset. **D** GS = 3 + 4 = 7/4 + 3 = 7 subset. GS = Gleason score.

Since the presence of cribriform cancer glands would be potentially useful in guiding management of low-intermediate-risk patients, we performed subset analysis using the 0.25 mm cutoff in several low-intermediate-risk population subsets. First, we evaluated the subset of patients with GS ≤ 3 + 4 = 7 carcinomas [Subset: GS ≤ 3 + 4 = 7 (i.e., combined Grade Groups 1 and 2), *N* = 867 patients]. In this GS ≤ 3 + 4 = 7 subset, the presence of cribriform glands of any size was not associated with adverse outcomes (univariable *p* = 0.126); however, when using the 0.25 mm size cutoff, the presence of cribriform glands with a size >0.25 mm was significantly associated with lower RFS (univariable *p* = 0.004, multivariable *p* = 0.018, Fig. 3A–B, Tables 2–3). Furthermore, in the GS ≤ 3 + 4 = 7 subgroup, patients with cribriform glands ≤0.25 mm performed similarly to patients without cribriform glands (Fig. 3C). Likewise, when we evaluated the subset of patients with GS 7 carcinomas [Subset: GS = 3 + 4 = 7/4 + 3 = 7 (i.e., combined Grade Groups 2 and 3), *N* = 496 patients], we also found that cribriform glands >0.25 mm was associated with lower RFS (univariable *p* = 0.004, multivariable *p* = 0.017, Tables 2–3, Fig. 3D), whereas cribriform glands alone was not a significant prognostic indicator (univariable *p* = 0.247, Table 2). Lastly, in the smaller subset of patients with GS 3 + 4 = 7 carcinoma [Subset: GS = 3 + 4 = 7 (i.e., Grade Group 2), *N* = 341 patients], patients with cribriform glands >0.25 mm showed a trend toward lower RFS that was not seen in patients with cribriform glands of any size; however, this finding did not reach statistical significance (univariable *p* = 0.1).

### Validation cohort

**Fig. 4:**
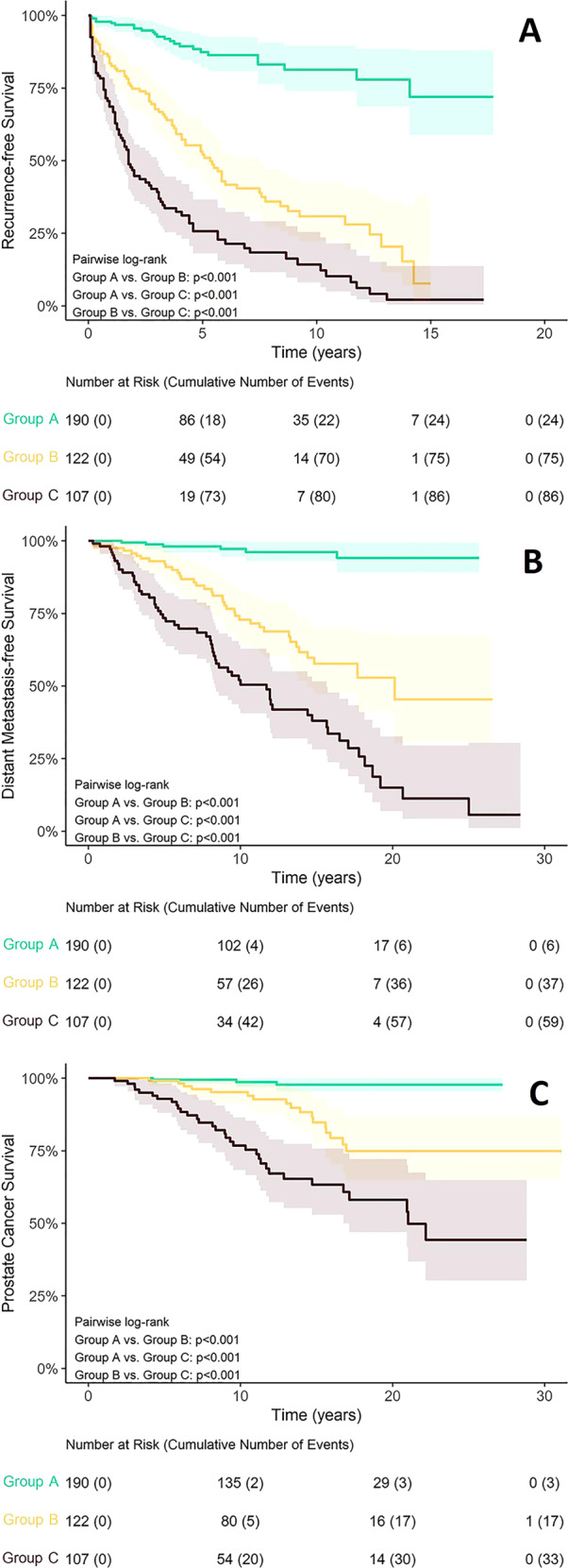
Kaplan-Meier curves for outcomes in validation cohort. **A** Biochemical recurrence. **B** Metastasis-free survival. **C** Prostate cancer survival.

A total of 419 patients had complete data available for assignment into the three groups of interest (Group A: without cribriform glands >0.25 mm or Gleason pattern 5; Group B: cribriform >0.25 mm and no Gleason pattern 5; and Group C: any Gleason pattern 5) and the outcome variables of interest. Median follow-up time for censored patients (patients who did not experience an event during the study observation period) was 13.5 years (range: 0.11–28.6 years). Median biochemical RFS and distant metastasis-free survival was 8.42 years and 25.0 years, respectively. Median prostate cancer survival was not reached during the observation period. On Kaplan–Meier analysis, patients with cribriform glands >0.25 mm had lower biochemical RFS (*p* < 0.001), metastasis-free survival (*p* < 0.001), and prostate cancer survival (*p* < 0.001) than patients without any cribriform glands or with cribriform glands ≤0.25 mm, even when cases with any admixed pattern 5 component were excluded (Fig. 4, Table 4). In addition, only 6 of 190 patients (3%) in Group A developed metastatic disease, compared to 37 of 122 (30%) in Group B and 59 of 107 (55%) in Group C. The 6 cases in Group A with metastatic disease all had Gleason pattern 4 architecture other than “small” cribriform glands, including 5 with anastomosing cords of carcinoma cells with variable lumen formation (Canary pattern Cz^6^) and one case with architecturally complex epithelium floating in confluent pools of mucin (Canary pattern Ew^6^). Of the 36 of 190 group A cases (19%) with small cribriform glands present (i.e., ≤0.25 mm), none developed metastases or had disease specific death.

**Table 4:** Validation cohort outcomes.

Outcome	Cribriform group	Number of subjects	Number of events	Median survival time (years)^a^	95% CI
Biochemical recurrence					
	Group A^b^	190	24	–	–
	Group B	122	75	5.3	(4.1, 7.6)
	Group C	107	86	1.8	(1.4, 3.1)
Distant metastasis					
	Group A^b^	190	6	–	–
	Group B	122	37	20.1	–
	Group C	107	59	11.7	(8.3, 15.7)
Prostate cancer death					
	Group A^b^	190	3	–	–
	Group B^b^	122	17	–	–
	Group C	107	33	21.0	–

Group A: No Gleason pattern 5; cribriform ≤ 0.25 mm or no cribriform.

Group B: No Gleason pattern 5; cribriform > 0.25 mm.

Group C: Any Gleason pattern 5.

^a^CI: Confidence Interval.

^b^Median survival was not reached during the observation period.

## Discussion

The cribriform growth pattern of prostatic adenocarcinoma is well-established as an adverse prognostic factor based on multiple measures of clinical outcome, including biochemical recurrence, associated metastatic disease, and disease specific survival^3,5–8,17,18^. Unfortunately, questions regarding optimal quantitative and qualitative histologic criteria for its diagnosis remain. The International Society of Urological Pathologists (ISUP) recently proposed the following definition for cribriform prostate cancer: “A confluent sheet of contiguous malignant epithelial cells with multiple glandular lumina that are easily visible at low power (objective magnification × 10). There should be no intervening stroma or mucin separating individual or fused glandular structures”^19^. This definition, as well as recent consensus grading papers by both ISUP and the GUPS, do not fully address the size of cribriform glands^1,2^. While evolving data on cribriform prostatic adenocarcinoma suggest that “large” or “expansile” cribriform morphology correlates strongest with aggressive behavior, authors have used varying criteria for classification, including cribriform growth with 12 or more luminal spaces, cribriform growth exceeding the size of an average benign gland, and cribriform growth at least twice the size of an average benign gland^3,5–7^. Although all previously proposed methods of evaluating cribriform size certainly overlap, it is difficult to compare micrometer measurements used in the current study to these other published criteria. To our knowledge, there are no formal studies that evaluate normal variation in diameter of prostate glands (particularly between regional anatomic zones of the prostate) or that correlate the number of luminal spaces with cribriform gland diameter. While the other proposed criteria are based on visual assessment of gland size and do not require actual measurement, it is our experience that use of a micrometer does not add excessive time to evaluating cribriform glands and is only necessary in borderline cases. For practical purposes, other internal controls can easily be used to assess relative size for very small and very large cribriform diameters (e.g., 0.25 mm is approximately half a microscopic field at × 400 magnification), precluding the need for ocular micrometer measurements in many cases.

Interestingly, a RP outcome study by Maru et al. that predates the resurgence in the cribriform literature, and is not usually referenced in cribriform reviews, reported that a diameter of perineural invasion >0.25 mm was a strong independent predictor of decreased progression free survival^20^. In our experience, perineural prostatic adenocarcinoma with “large diameter” is exclusively cribriform in architecture. Based on the images provided from this study and one other, we hypothesized that the diameter of perineural invasion is likely a surrogate of large diameter cribriform gland morphology^20,21^. Indeed, statistical analysis of our data identified exactly the same optimal prognostic cut-off of 0.25 mm.

We also performed analyses on multiple low-intermediate risk subsets in the training cohort, as there is increasing need for additional predictive biomarkers to guide treatment strategies in this sub-population. NCCN guidelines lists active surveillance as a recommended initial management strategy for patients with “favorable intermediate-risk” prostate cancer, defined in part by patients with Grade Group 1 or 2 (GS 3 + 3 = 6, PSA 10–20; or 3 + 4 = 7) on biopsy; however, widespread adoption of active surveillance for this patient population has been limited^22^. Our results support the findings of Hollemans et al. that large cribriform growth (using a definition of diameter at least twice the size of adjacent preexistent normal glands) is an independent predictor of biochemical recurrence in a RP cohort comprised of ISUP Grade Group 2 (GS 3 + 4 = 7) disease^5^. Both our current data and those of Hollemans et al. are in contrast to the findings of Keefe et al. and Iczkowski et al.^3,4^. The varying results are very likely due to very low numbers of small cribriform glands in one study (i.e., likely underpowered), different criteria utilized for the distinction of “small” and “large” cribriform glands, and the inherent differences between cohorts with regard to the outcome standard utilized (i.e., prediction of grade and stage at definitive surgery vs clinical outcome measures). These differences further underscore the importance of identifying an outcome-based measure for a definition of large cribriform glands.

We validated the 0.25 mm cut-off in a second separate whole section RP cohort, in which all RPs were completely embedded, and all histologic sections were re-reviewed for evaluating histologic patterns. We evaluated cases with any conventional Gleason pattern 5 separately to exclude the possibility of a conventionally higher-grade component driving prognosis. The biochemical free recurrence, metastasis-free survival, and prostate cancer survival were significantly lower for tumors with “large” cribriform pattern compared to those without (i.e., either no cribriform or cribriform ≤0.25 mm). In fact, only six patients (3%) in the group with no “large” cribriform pattern and no pattern 5 had metastatic disease. Interestingly, each of these tumors had other Gleason pattern 4 subtypes not fitting the standard definition of cribriform but previously described as “high-risk” by the original Canary histology study^6^. These include Canary patterns Cz (*n* = 5) and Ew (*n* = 1), which by histologic description are complex anastomosing cords with variable lumen formation and complex epithelium floating in confluent pools of mucin, respectively. In our experience, these patterns do not commonly represent the only high-risk pattern in a tumor, which is likely why the study of cribriform architecture has dominated the literature as the aggressive subtype of Gleason pattern 4 disease. Most importantly, we did not identify any cases with “small’” cribriform glands (i.e., ≤0.25 mm) as the most complex architectural pattern that developed metastatic disease.

There are limitations of this study. First, in the training cohort, a small percentage of the cases classified as GS 3 + 3 = 6 upon central review (by JKM) were classified as containing cribriform glands (by EC) [30 of 1287 in overall cohort (2%); 30 of 526 in 3 + 3 = 6 subset (6%)]. This is not entirely unexpected given the known interobserver variability in the minimal threshold required for diagnosis as “small” cribriform glands^19,23,24^. The discrepant cases were either classified as cribriform ≤0.25 mm or likely “borderline” cribriform glands at the threshold for diagnosis (i.e., complex gland architecture associated with mucin, early lesions with few intraluminal spaces, or glomeruloid patterns). Regardless of classification as cribriform or non-cribriform, patients with these borderline patterns would still be appropriately placed in the low-risk group and should not have affected the results of the study. Moreover, the existence of such borderline cases makes an even stronger argument for requiring a quantitative measurement for diagnosis of large cribriform glands, which in our experience would exclude most discrepant cases. In addition, the outcome measure for the training cohort is RFS, which includes PSA-defined biochemical recurrence. While biochemical recurrence is a standard measure used in prostate cancer research, its limitations as an outcome surrogate are well recognized and previously described^25–28^. We had previously addressed the design of our TMA and potential sampling bias in our original manuscript^6^. The highest Gleason pattern foci were sampled by three cores, which should limit issues with missing a high-risk pattern. Furthermore, our original study showed that the cohort could demonstrate differences in outcome based on the presence of certain architectural patterns of adenocarcinoma, including cribriform glands. This training cohort was carefully constructed by a statistician (ZF) to increase statistical power for identifying features that outperform conventional Gleason grading^9^. Specifically, low grade carcinomas (GS 6; Grade Group 1) with recurrence and high grade carcinomas (GS 8–10; Grade Group 4–5) without recurrence were over-selected. Because of this, the recurrence data would not mirror expected outcomes for consecutive series or a population-based study. By design these intentional biases should have made finding statistical differences for variables that co-vary with grade more difficult. Moreover, the validation cohort addresses many of these limitations as it utilized cases in which histologic patterns were completely scored in every section of totally embedded RPs, and for which long-term metastasis-free survival and prostate cancer survival could be used as endpoints.

This study does not address biopsy management. The identification of large cribriform glands on biopsy is known to suffer from sampling error, and the sensitivity of core needle biopsy for identifying large cribriform carcinoma at RP is ~45–60%^29–31^. Therefore, other adjunctive tests (e.g., imaging, serum biomarkers, or genomic classifiers) may have a role in predicting for unsampled large cribriform gland morphology (or other high-risk patterns) for optimal active surveillance management. Small cribriform glands are often admixed with large cribriform glands; therefore, the identification of small cribriform glands on biopsy could potentially be predictive for an increased risk of unsampled large cribriform glands. Such questions will require further study utilizing a paired biopsy and RP cohorts.

Our study, like many, does not address subclassification as intraductal carcinoma (IDC-P), which can manifest as cribriform glands lined by basal cells and therefore shares overlapping features with invasive cribriform Gleason pattern 4 carcinoma. Incorporation of IDC-P into Gleason grading is currently controversial due to the two methods in which IDC-P can arise: as a form of in situ carcinoma or as retrograde involvement of invasive cancer into the prostatic glands/ducts, the latter of which is thought to be much more common and particularly aggressive form of IDC-P^32^. In this study, we combined and measured all cribriform tumor glands, IDC-P and invasive, for the following reasons: IDC-P and invasive cribriform Gleason pattern 4 are often morphologically indistinguishable, basal cell markers are not routinely performed in clinical practice to evaluate for IDC-P, and several studies have shown that intraductal carcinoma behaves like invasive carcinoma and should be graded as such^2,11–13^.

In conclusion, our recurrence free survival-based outcome analysis of a 1287 patient retrospective RP cohort identified >0.25 mm as the optimal size criterion for “large” cribriform prostatic adenocarcinoma. This definition validated in a second RP cohort for each outcome measure tested: biochemical free recurrence, metastasis-free survival, and prostate cancer survival. Finally, we confirm the significant metastatic risk associated with “large” cribriform morphology independent of Gleason pattern 5 disease.

## Data Availability

The datasets used and/or analyzed during the current study are available from the corresponding author on reasonable request.
